# MiR-146b overexpression promotes bladder cancer cell growth via the SMAD4/C-MYC/Cyclin D1 axis

**DOI:** 10.3389/fonc.2025.1565638

**Published:** 2025-03-28

**Authors:** Junlan Zhu, Zhijian Zheng, Zhangya Yin, Linchao Ding, Congya Li, Xuyao Wang, Peng Shu, Jun Zhou, Weihua Liu, Jian Liu

**Affiliations:** ^1^ Precision Medicine Laboratory, Beilun People’s Hospital, Beilun Branch of the First Affiliated Hospital, School of Medicine, Zhejiang University, Ningbo, Zhejiang, China; ^2^ Department of Medical Oncology, Affiliated Jinhua Hospital, Zhejiang University School of Medicine, Jinhua, China; ^3^ Department of Scientific Research, Affiliated Jinhua Hospital, Zhejiang University School of Medicine, Jinhua, China; ^4^ Department of Urology, Beilun People’s Hospital, Beilun Branch of the First Affiliated Hospital, School of Medicine, Zhejiang University, Ningbo, Zhejiang, China

**Keywords:** MiR-146b, bladder cancer cell growth, SMAD4, C-MYC, Cyclin D1

## Abstract

MiR-146b has been identified as being overexpressed in human bladder cancer (BCa) and implicated in promoting cancer cell invasion. However, its specific involvement in BCa cell growth remains unclear. In this study, we demonstrate that the downregulation of miR-146b significantly suppresses tumorigenic growth of human BCa cells both *in vitro* and *in vivo* by inducing G0/G1 cell cycle arrest. Specifically, miR-146b inhibition resulted in a significant reduction in colony formation (p < 0.05) and anchorage-independent growth in both UMUC3 and T24T cells, as measured by soft agar assays, with three independent replicates for each experiment. Notably, Cyclin D1 protein plays a crucial role in miR-146b-induced BCa cell proliferation, as confirmed by Western blotting (p < 0.05), with each experiment performed in triplicate. Mechanistic investigations reveal that miR-146b reduces mothers against decapentaplegic homolog 4 (SMAD4) mRNA stability by directly binding to its 3′ untranslated region (3′-UTR), leading to decreased SMAD4 expression. This reduction in SMAD4 levels promotes cellular myelocytomatosis (C-MYC) transcription, which in turn enhances Cyclin D1 transcription, ultimately facilitating BCa cell proliferation. The findings unveil a novel regulatory axis involving SMAD4/C-MYC/Cyclin D1 in mediating the oncogenic role of miR-146b in BCa cells. Statistical significance was determined using Student’s t-test, with p-values <0.05 considered significant. Together with its previously established function in BCa invasion, the results highlight the potential for developing miR-146b-based therapeutic strategies for treating human BCa patients.

## 1 Introduction

Bladder cancer (BCa) is the most common malignant tumor of the urinary system. The mortality rate of early-stage bladder cancer is not very high, but after lymphatic metastasis, most patients survive only approximately 2 years (1). Bladder cancer can be divided into two types, non-muscle-invasive bladder cancer (NMIBC) and muscle-invasive bladder cancer (MIBC) (2). NMIBC is treated by transurethral resection of bladder cancer, followed by intravesical treatment. However, up to two-thirds of the tumor patients will experience recurrence or progress to MIBC. For MIBC patients who have received radical cystectomy or systematic chemotherapy, the 5-year survival rate is still less than 60% (3). Thus, it is urgently needed to study the new biomarkers for the early detection and targeting of bladder cancer.

MicroRNAs (miRNAs), highly conserved small non-protein coding RNAs, participate in maintaining cell homeostasis by regulating gene expression (4). Proper regulation of miRNA expression is important because miRNAs influence almost every signaling pathway involved in cell proliferation, cell motility, and cell self-renewal (5). Deregulation of miRNA expression is observed in multiple tumors by playing the role of tumor suppressors or oncogenes (6). The oncogenic role of miR-146b has been identified in various malignancies, including cervical (7), colorectal (8), and prostate cancers (9). Our previous study also demonstrated that miR-146b was upregulated in human BCa tissues and cells. MiR-146b overexpression promoted the invasive ability of BCa cells by increasing *mmp2* mRNA transcription and protein expression (10). However, the mechanism underlying the roles of miR-146b regulating bladder cancer tumorigenic growth remains poorly understood. MiR-146b was chosen for this study due to its well-established role as an oncogenic microRNA in various cancers, including bladder cancer, where it has been shown to promote tumor invasion and progression. Previous studies have highlighted the upregulation of miR-146b in human bladder cancer tissues and its involvement in enhancing the invasive properties of bladder cancer cells by regulating matrix metalloproteinases (MMPs). Additionally, miR-146b has been implicated in the regulation of several critical cancer-related pathways, making it an attractive candidate for further exploration in the context of bladder cancer. While miR-146b holds great promise as a therapeutic target in bladder cancer, it is important to consider potential off-target effects and the challenges associated with targeting miRNAs. MiRNAs often regulate multiple genes involved in various biological processes, and their inhibition or overexpression can lead to unintended effects on non-target pathways. For example, miR-146b is known to interact with several target genes involved in inflammation, immunity, and other cancer-related pathways. As a result, therapies targeting miR-146b may have broader biological consequences that need to be carefully evaluated. Additionally, the delivery of miRNA-based therapies remains a significant challenge due to the need for efficient, specific, and safe delivery systems. Furthermore, there is a risk of immune system activation or off-target gene silencing due to the broad spectrum of miRNA–target interactions. Future studies should focus on optimizing the delivery mechanisms for miRNA-based therapies, minimizing off-target effects, and developing strategies to selectively modulate miR-146b expression in cancer cells. In addition to miR-146b, several other miRNAs have been shown to target key components of the SMAD4/C-MYC/Cyclin D1 axis. For example, the miR-17-92 cluster (11), including miR-17 and miR-20a, has been implicated in modulating the TGF-β signaling pathway by targeting SMAD2, SMAD4, and TGFBR2, contributing to cancer progression, and miR-26a (12) has been identified as a tumor suppressor in multiple cancers, including hepatocellular carcinoma, by directly targeting Cyclin D2 and Cyclin E2, leading to G1 phase cell cycle arrest. These miRNAs can regulate the SMAD4/C-MYC/Cyclin D1 axis through various mechanisms, influencing cell proliferation, invasion, and metastasis. While the role of miR-146b in bladder cancer remains under investigation, other miRNAs targeting this axis further highlight the potential of targeting miRNA pathways as a therapeutic strategy in cancer treatment.

Acting as the regulatory subunit of the cyclin-dependent kinases (CDKs), Cyclin D1 is sequentially expressed and integrates extracellular signals with the cell cycle machinery to drive cell cycle G1–S phase transition and cellular proliferation (13). The Cyclin D1 gene is regarded as the oncogene, the amplifications of which have been detected in cancers of various organs. Our group found that Cyclin D1 downregulation was responsible for the isorhapontigenin-induced anticancer effect on human bladder cancer cells (14). Transcriptional coordination of cellular myelocytomatosis (C-MYC) with Cyclin D1 was reported to accelerate tumor formation and make the tumor progress into a more aggressive phenotype (15). Moreover, acting as the upstream activator of Cyclin D1, C-MYC may elicit the transformation of cancer cells. Thus, targeting C-MYC and Cyclin D1 will be of great significance for the prevention and treatment of BCa. CDKs play a crucial role in regulating the cell cycle, particularly in driving the G1 to S phase transition, which is essential for cellular proliferation. These kinases are activated by cyclins, with Cyclin D1 being a key regulator of the G1 phase. Amplifications of the Cyclin D1 gene are commonly observed in cancers, where its upregulation drives unregulated cell growth. C-MYC, a transcription factor, acts as an upstream activator of Cyclin D1, promoting tumor progression by accelerating the cell cycle and enhancing cell proliferation. MiR-146b, an oncogenic microRNA, has been implicated in various malignancies, including bladder cancer, where it promotes tumor progression by regulating genes involved in cell invasion and matrix remodeling, such as MMP2. Additionally, miR-146b may influence Cyclin D1 and C-MYC expression, further contributing to the proliferative and invasive properties of cancer cells. Together, Cyclin D1, C-MYC, and miR-146b form an interconnected regulatory network that drives the rapid proliferation and progression of bladder cancer, making them potential targets for therapeutic intervention.

The SMAD4/C-MYC/Cyclin D1 axis plays a pivotal role in regulating key aspects of cancer biology, particularly in the context of cell cycle progression, proliferation, and tumor growth. SMAD4 is a key mediator of the TGF-β signaling pathway, which is traditionally known for its tumor-suppressive functions, including the induction of cell cycle arrest and apoptosis in normal cells. However, in cancer, the loss or downregulation of SMAD4 can disrupt these processes, leading to uncontrolled cell proliferation. This disruption often results in the activation of downstream oncogenes, including C-MYC, which promotes the transcription of Cyclin D1, a critical regulator of the G1–S phase transition. Overexpression of Cyclin D1 is commonly observed in various cancers, where it accelerates cell cycle progression and contributes to tumorigenesis. The interplay between SMAD4, C-MYC, and Cyclin D1 thus forms an essential signaling network that governs the proliferative capacity of cancer cells. In this study, we propose that miR-146b-induced repression of SMAD4 leads to enhanced C-MYC expression and subsequent upregulation of Cyclin D1, facilitating the rapid growth and metastasis of bladder cancer cells. Understanding this axis not only offers insights into the molecular mechanisms underlying cancer progression but also presents potential therapeutic opportunities for targeting this pathway in cancer treatment.

BCa remains one of the most prevalent malignancies of the urinary system, characterized by a high recurrence rate and poor prognosis, particularly in muscle-invasive cases. Despite advancements in diagnostic and therapeutic strategies, the molecular mechanisms driving BCa progression remain insufficiently understood. MiRNAs, small non-coding RNAs, play a crucial role in regulating gene expression and are involved in various cellular processes including cell proliferation, invasion, and metastasis. MiR-146b has been identified as an oncogene in several cancers, including bladder cancer, where it is implicated in promoting cell invasion through the regulation of matrix metalloproteinases. However, its specific role in the regulation of BCa cell growth and tumorigenesis remains unclear. In this context, we explore the significance and therapeutic potential of miR-146b in bladder cancer. We will investigate the oncogenic role and the molecular mechanisms of miR-146b on human BCa cell proliferation and anchorage-independent growth. The objective of this study was to investigate the oncogenic role of miR-146b in bladder cancer cell growth and to explore the underlying molecular mechanisms. Specifically, we aimed to determine how miR-146b influences key cell cycle regulators, such as Cyclin D1, and the associated molecular pathways involving SMAD4, C-MYC, and Cyclin D1. By understanding these mechanisms, we seek to provide new insights into the regulatory networks that drive BCa cell proliferation, with the ultimate goal of identifying miR-146b as a potential therapeutic target for bladder cancer treatment.

## 2 Materials and methods

### 2.1 Plasmids, antibodies, and reagents

The aim of the experiment was to investigate the role of miR-146b inhibitor and its nonsense control in regulating various transcription factors and signaling pathways, particularly Cyclin D1, c-Myc, and SMAD4. The lentiviral constructs of miR-146b inhibitor and its nonsense control were constructed by GenePharma (Shanghai, China). The plasmid of the human Cyclin D1 promoter (from −1,407 to −167)-driven luciferase reporter was constructed with *Xho*I and *Hin*dIII using genomic DNA purified from UMUC3 cells based on the National Center for Biotechnology Information (NCBI) database. The plasmid of the human c-myc promoter (from −1,225 to −14)-driven luciferase reporter was constructed with *Xho*I and *Hin*dIII using genomic DNA purified from UMUC3 cells based on the NCBI database. The human mothers against decapentaplegic homolog 4 (smad4) 3′ untranslated region (3′-UTR) was cloned into the pMIR-report luciferase vector through the *Spe*I and *Hin*dIII sites. Smad4 3′-UTR point mutation was amplified from the WT template by overlap PCR using the following primers: forward, 5′-TTTAAAGGCAGAGAAGCCATCAAAGTTAATTCA-3′; reverse, 5′-TGAATTAACTTTGATGGCTTCTCTGCCTTTAAA-3′. The HA-tagged Cyclin D1 constitutively expressed plasmid and pT3EF1a-C-MYC plasmid were obtained from Addgene (Cambridge, MA, USA). The constructs of short hairpin RNA specifically targeting SMAD4 (shSMAD4) were purchased from Santa Cruz Biotechnology (Santa Cruz, CA, USA). All plasmids were prepared by the Plasmid Preparation/Extraction Maxi Kit from QIAGEN (Valencia, CA, USA). The antibodies specific against CDK4, CDK6, Cyclin D1, Cyclin E1, p27, HA, c-Jun, C-MYC, Elk1, ETS1, Sp1, SMAD4, GAPDH, and β-actin were bought from Cell Signaling Technology (Beverly, MA, USA). Actinomycin D (Act D) was bought from Calbiochem (Billerica, MA, USA).

### 2.2 Cell culture and transfection

The purpose of this experiment was to investigate the effects of specific gene modifications on human BCa cell lines T24T and UMUC3. The human BCa cell lines T24T and UMUC3 were used in the study. All cancer cell lines were subjected to DNA tests and authenticated before use for the studies. UMUC3 cells were maintained at 37°C in a 5% CO_2_ incubator in Dulbecco’s modified Eagle’s medium (DMEM) (Gibco, Grand Island, NY, USA) supplemented with 10% fetal bovine serum (FBS) (Gibco, Grand Island, NY, USA). T24T cells were cultured with a 1:1 mixture of DMEM/Ham’s F12 medium (Gibco, Grand Island, NY, USA) supplemented with 5% FBS. Stable transfections were performed with constructs using PolyJet DNA *In Vitro* Transfection Reagent (SignaGen Laboratories, Gaithersburg, MD, USA) according to the manufacturer’s instructions, and stable transfectants were selected with puromycin (0.2–0.3 mg/mL) or hygromycin B (200–400 mg/mL) for 3 or 4 weeks according to the different antibiotic resistance plasmids transfected (16, 17). Although the cell lines and animal models used in this study are widely accepted as representative models for human bladder cancer, it is important to acknowledge their limitations. The UMUC3 and T24T human bladder cancer cell lines, while commonly used in bladder cancer research, may not fully recapitulate the heterogeneity of human tumors, particularly in terms of the tumor microenvironment and metastasis. Additionally, these cell lines are derived from advanced stages of bladder cancer, which may not completely represent early-stage disease or the full spectrum of bladder cancer subtypes.

### 2.3 Protein expression analysis of BCa cells and transfectants

The aim of this experiment was to analyze the protein expression of specific targets in BCa cells and transfectants using Western blotting analysis. As previously described (10), BCa cells and the transfectants were seeded in six-well plates and cultured in a normal culture medium until 70%–80% confluence. The whole cell extracts were prepared and were then subjected to Western blotting analysis. The images were acquired by scanning with ChemiDoc™ MP Imaging System from Bio-Rad (Hercules, CA, USA).

### 2.4 Analysis of miR-146b and target gene expression in cultured cells

The aim of this experiment was to measure the expression levels of specific genes and miRNAs in BCa cells and transfectants using quantitative real-time PCR (qRT-PCR). First, the RNA was extracted from the cultured cells using TRIzol reagent (Invitrogen, Carlsbad, CA, USA). Reverse transcriptase was used to produce the first-strand complementary DNA (Aidlab, Beijing, China) according to the manufacturer’s instructions. MiRNA real-time PCR Assay kit was used to detect the expression level of miR-146b (Aidlab, China). Furthermore, U6 was chosen to be the internal control. The primers used in this study were as follows: miR-146b (forward, 5′-TGA CCC ATC CTG GGC CTC AA-3′; reverse, 5′-CCA GTG GGC AAG ATG TGG GCC-3′), U6 (forward, 5′-CTC GCT TCG GCA GCA CA-3′; reverse, 5′-AAC GCT TCA CGA ATT TGC GT-3′), human Cyclin D1 (forward, 5′-GAGGTCTGCGAGGAACAGAAGTG-3′; reverse, 5′-GAGCGGGGATTGGAAATGAACTTC-3′), human C-MYC (forward, 5′-AAC ACA CAA CGT CTT GGA GC-3′; reverse, 5′-CCT TAC GCA CAA GAG TTC CG-3′), human SMAD4 (forward, 5′-ACA AGT AAT GAT GCC TGT CTG A-3′; reverse, 5′-CTC CCA TCC AAT GTT CTC TGT A-3′), and human GAPDH (forward, 5′-GAT GAT CTT GAG GCT GTT GTC-3′; reverse, 5′-CAG GGC TGC TTT TAA CTC TG-3′). The qRT-PCR analysis was carried out using the SYBR Green PCR kit (Qiagen, Santa Clarita, CA, USA) and the 7900HT Fast Real-time PCR system (Applied Biosystems, Carlsbad, CA, USA). The ΔΔCT value was used to calculate the relative expression of the indicated mRNA using gapdh as an endogenous control.

### 2.5 Luciferase assay

The aim of this experiment was to evaluate the luciferase activity of human Cyclin D1, c-myc promoters, and SMAD4 3′-UTR in BCa cells with different treatments. For the determination of human cyclin d1 promoter-driven luciferase activity and human c-myc promoter-driven luciferase activity, the indicated cells were each transiently co-transfected with pRL-TK, together with the related promoter-driven luciferase reporter. Twenty-four hours after transfection, luciferase activity was determined using the Luciferase Assay System Kit (Promega, Madison, WI, USA). For the determination of smad4 mRNA 3′-UTR activity, UMUC3 (nonsense) and UMUC3 (miR-146b inhibitor) cells were transiently transfected with pRL-TK together with smad4 mRNA 3′-UTR luciferase reporter. Twenty-four hours after transfection, luciferase activity was determined using the Luciferase Assay System Kit (Promega, Madison, WI, USA). The results were normalized by internal TK signal. All experiments were conducted in triplicate, and the results were expressed as mean ± standard error.

### 2.6 ChIP assay

The aim of this experiment was to investigate the binding of specific transcription factors to the human c-myc promoter using chromatin immunoprecipitation (ChIP) assay. ChIP assay was carried out as described in previous publications using reagents that were purchased from Millipore Technologies (Billerica, MA, USA) (18). To specifically amplify the region containing the putative responsive elements on the human c-myc promoter, PCR was performed with the following pair of primers: forward, 5′-GAG AAA TTG GGA ACT CCG TG-3′; reverse, 5′-CAA AGC AGC AGA TAC CGC CC-3′. The PCR products were separated on 2% agarose gels and stained with ethidium bromide; the images were then scanned using a UV light.

### 2.7 Cell proliferation analysis

The aim of this experiment was to assess the cell viability of BCa cells under different conditions using the CellTiter-Glo Luminescent Cell Viability Assay. Cell viability was determined by utilizing the CellTiter-Glo Luminescent Cell Viability Assay Kit (Promega, Madison, WI, USA) according to the manufacturer’s instructions. Briefly, cells were plated in 96-well plates at a density of 5,000 cells per well and allowed to adhere overnight. The cell culture medium was replaced with 0.1% FBS DMEM, cultured for 12 h, then replaced with a normal medium, and cultured for another 1, 2, 3, or 4 days, and then 50 μL CellTiter-Glo assay reagent was added to each well. The contents were mixed on an orbital shaker for 2 minutes to induce cell lysis and then incubated at room temperature for 10 minutes to stabilize the luminescent signal. Results were read on a microplate luminometer LB 96V (Berthold GmbH & Co. KG, Bad Wildbad, Germany). Cell viability (%) was defined as the relative absorbance of treated samples versus that of the untreated control. All experiments were performed in 96-well plates for each experiment and repeated at least three times.

### 2.8 Cell cycle analysis

The aim of this experiment was to analyze the cell cycle distribution of BCa cells under different conditions using flow cytometry with the BD Cycletest™ Plus DNA Reagent kit. The indicated cells (2 × 10^5^) were cultured in each well of 6-well plates to 70%–80% confluence with normal culture medium. Following serum starvation for 12 h, the medium was replaced with 10% FBS DMEM for another 12 h. Then, the cells were treated with the BD Cycletest™ Plus DNA Reagent kit to treat the cultured cells (BD Biosciences, San Jose, CA, USA). First, the cell debris and fixation artifacts were gated out. Subsequently, the cell populations were quantified at the G0/G1, S, and G2/M phases using the ModFit software.

### 2.9 Anchorage-independent growth assay

The aim of this experiment was to assess the anchorage-independent growth of BCa cells by performing a soft agar colony formation assay. The 1 × 10^4^ cells in 10% FBS Basal Medium Eagle (BME) containing 0.33% soft agar were seeded over the basal layer containing 0.5% agar containing 10% FBS BME in each well of 6-well plates. The plates were incubated in a 5% CO_2_ incubator at 37°C for 3 weeks. Colonies were captured under a microscope, and only colonies with over 32 cells were counted. The results were presented as mean ± SD obtained from three independent experiments.

### 2.10 Xenograft model in nude mice *in vivo*

The aim of this experiment was to evaluate the *in vivo* tumorigenic potential of UMUC3 cells with miR-146b inhibitor or nonsense control in BALB/c athymic nude mice. BALB/c athymic nude mice (3–4 weeks old) were purchased from Vital River (Beijing, China). After 2-week acclimatization, the mice were randomly allocated into two groups (five in each group) and subcutaneously injected in the right side (lower back) with 0.1 mL UMUC3 (miR-146b inhibitor) and UMUC3 (nonsense) cells [in each case, 2 × 10^6^ cells suspended in 100 μL phosphate-buffered saline (PBS)]. The sample size for the *in vivo* xenograft experiments was determined based on power calculations to ensure adequate statistical power for detecting significant differences in tumor growth between the experimental and control groups. A total of five mice per group (n = 5) were included, which is consistent with standard practices in similar studies involving xenograft tumor models. This sample size was selected to balance statistical power with ethical considerations and animal welfare. Tumor volumes and weights were measured weekly, and differences between groups were analyzed for statistical significance. After 4–5 weeks, the mice were sacrificed, and any tumor present was surgically removed, imaged, and weighed. The study for the animals was approved by the Experimental Animal Welfare and Ethics Committee of Affiliated Jinhua Hospital, Zhejiang University School of Medicine (Approval No. AL-JHYY202345). While athymic nude mice are useful for xenograft studies, they lack a fully functional immune system, which limits the ability to assess the role of immune responses in cancer progression and treatment. Future studies using primary bladder cancer samples, patient-derived xenografts (PDXs), or genetically engineered mouse models (GEMMs) could provide more accurate representations of tumor biology and therapeutic responses. Despite these limitations, the models used in this study are well-established tools that provide valuable insights into the molecular mechanisms driving bladder cancer growth and progression.

### 2.11 Statistical analysis

Statistical analysis was performed using the Prism 5.0 software (GraphPad Software, San Diego, CA, USA). All data were presented as the means of triplicate assays ± SD. Student’s t-test was employed to determine the significance of differences between various groups. The differences were considered significant at p < 0.05.

## 3 Result

### 3.1 MiR-146b overexpression promoted human BCa tumorigenic growth both *in vitro* and *in vivo*

**Figure 1:**
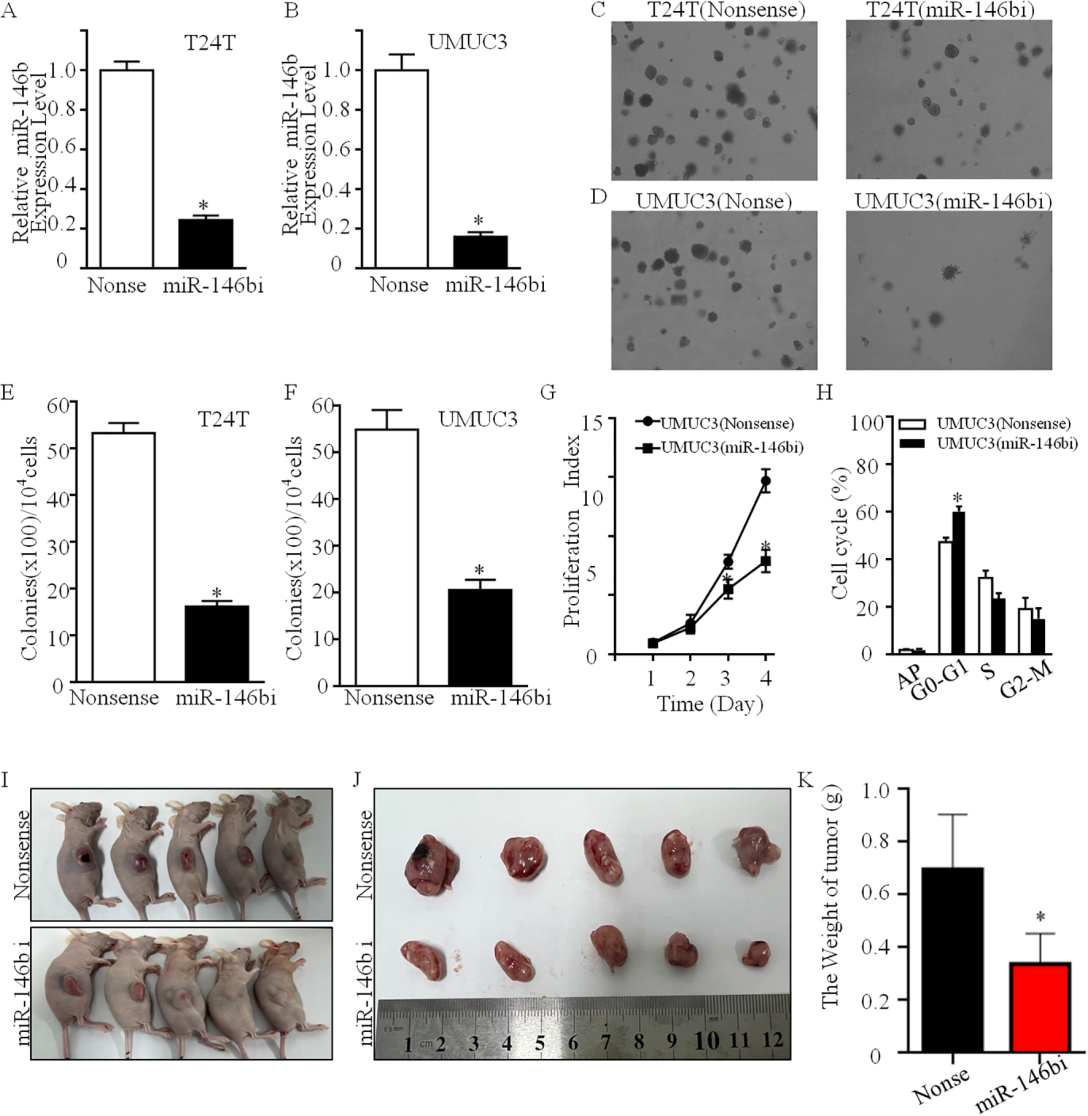
MiR-146b overexpression was crucial for anchorage-independent growth of human BCa cells. **(A, B)** MiR-146b inhibitor and its nonsense control plasmids were stably transfected into T24T **(A)** and UMUC3 **(B)** cells, and stable transfectants were identified by real-time PCR. Bars represent the mean ± SD, Student’s *t*-test was used to determine the p-value, and the asterisk (*) indicates a significant decrease relative to nonsense control cells (*p < 0.05). **(C, D)** A soft agar assay was used to determine the effect of miR-3687 down-expression on UMUC3 and T24T anchorage-independent growth, and the images were captured under microscopy. **(E, F)** The number of colonies was counted with more than 32 cells of each colony, and the results were presented as colonies per 10,000 cells, and the bars show mean ± SD from three independent experiments. The asterisk (*) indicates a significant decrease in comparison to that of nonsense control transfectants (*p < 0.05). **(G)** UMUC3 transfectants were plated in 96-well plates at a density of 5,000 cells per well. The cell culture medium was then replaced with 0.1% FBS DMEM and cultured for 12 h The cells were replaced with normal medium and cultured for another 1, 2, 3, or 4 days. Subsequently, an ATP activity assay was performed using protocol described in the section “Materials and Methods”. The double asterisk (*) indicates significant decrease from nonsense control (p < 0.05). **(H)** The indicated cells (2 × 10^5^) were seeded into a 6-well plate and cultured overnight. Following synchronization in 0.1% FBS for 12 h, the medium was replaced with 10% FBS DMEM for another 12 h, and the cells were then stained with propidium iodide for cell cycle analysis as described in the section of “Materials and Methods”. The results represented one of three independent experiments. BALB/c athymic nude mice were subcutaneously injected with UMUC3 (miR-146bi) cells or their nonsense control transfectants. The mice were euthanized after 4–5 weeks, and xenograft tumors in the mice injected with the indicated UMUC3 transfectants were removed, imaged, and weighed **(I, J)**. The results of the tumor weight in the mice injected with the UMUC3 transfectants **(K)** were presented as means ± SD. *Significant decrease relative to nonsense control cells (p < 0.05). BCa, bladder cancer; FBS, fetal bovine serum; DMEM, Dulbecco’s modified Eagle’s medium.

Our recent study has discovered that miR-146b is highly expressed in human BCa tissues and cells, which plays a promotion role in BCa cell invasion by upregulating MMP2 protein expression (10). To evaluate the biological role of miR-146b in regulating BCa cell proliferation and tumorigenic growth, the inhibitor constructs of miR-146b were stably transfected into UMUC3 and T24T human BCa cells (p < 0.05), respectively (**Figures 1A, B**). Colony formation assay results showed that miR-146b inhibition decreased the anchorage-independent growth in both T24T and UMUC3 cells (**Figures 1C–F**).

Moreover, the inhibition of miR-146b significantly reduced the monolayer growth of UMUC3 and T24T, accompanied by inducing G0/G1 cell cycle arrest in UMUC3 cells (**Figures 1G, H**). To further determine the potent oncogenic activity of miR-146b *in vivo*, UMUC3 (miR-146b inhibitor) and UMUC3 (nonsense) cells were subcutaneously injected into nude mice. Unexpectedly, the inhibition of miR-146b dramatically decreased UMUC3 xenograft tumor growth and reduced tumor burden (weight) as compared to the UMUC3 (nonsense) group (p < 0.01, n = 5) (**Figures 1I–K**). In general, a novel positive regulatory effect of miR-146b on human BCa cell growth has been discovered.

### 3.2 Cyclin D1 protein mediated miR-146b-induced human BCa cell proliferation

**Figure 2:**
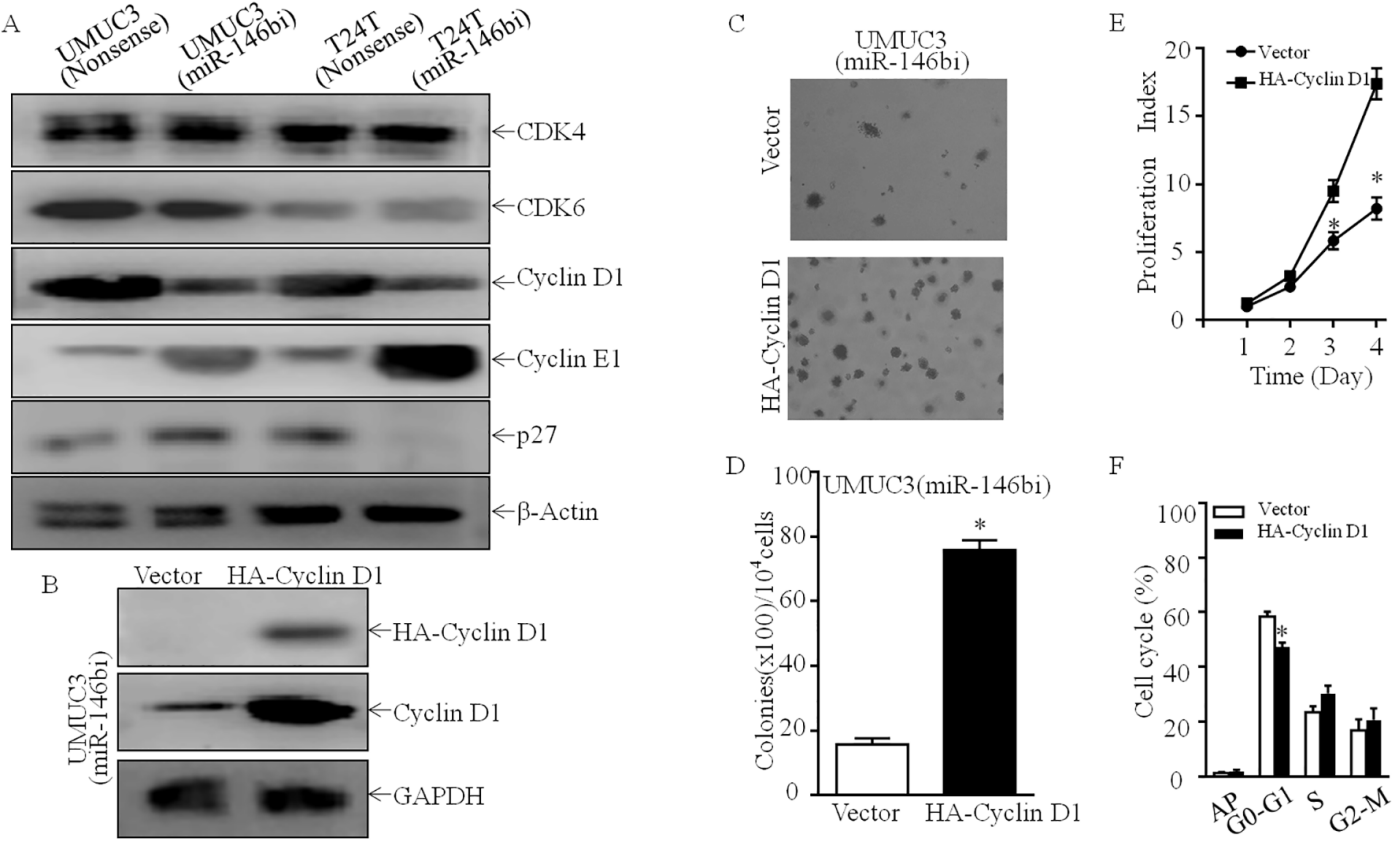
Cyclin D1 upregulation mediates miR-146b promotion of human BCa anchorage-independent growth. **(A)** The indicated cell extracts were subjected to Western blotting to determine the expression of CDK4, CDK6, Cyclin D1, Cyclin E1, and p27. β-Actin was used as a protein loading control. **(B)** HA-tagged Cyclin D1 constructs were stably transfected into UMUC3 (miR-146bi) cells. The overexpressed efficiency of Cyclin D1 protein was assessed by Western blotting. **(C, D)** A soft agar assay was used to determine the effect of Cyclin D1 overexpression on anchorage-independent growth of UMUC3 (miR-146bi); the number of colonies was counted under microscopy **(C)**, and the results were presented as colonies per 10,000 cells **(D)**. **(E)** The effect of Cyclin D1 on monolayer proliferation rates of UMUC3 (miR-146bi) cells was evaluated by ATP assay. Results are means ± SD; *significant change relative to vector control cells (p < 0.05). **(F)** The indicated cells were seeded in 6-well plates and cultured to 70%–80% confluence. After synchronization, the cells were cultured in complete medium for another 24 h and then subjected to cell cycle analysis (n = 3). BCa, bladder cancer.

In order to elucidate the mechanism of miR-146b involved in the regulation of G0/G1 cell cycle transition, we first performed Western blotting to detect the potential G0/G1 transition regulators. As the result showed, the knockdown of miR-146b exerted no remarkable effect on CDK4, CDK6, and p27 protein expression, while specifically decreasing Cyclin D1 protein abundance in both UMUC3 and T24T cells (**Figures 2A, B**). Therefore, we supposed that Cyclin D1 may be the miR-146b downstream effector involved in inducing G0/G1 phase arrest and promoting human BCa cell proliferation. To test this notion, we stably transfected HA-Cyclin D1 into UMUC3 (miR-146bi) cells (**Figure 2B**). The ectopic expression of HA-Cyclin D1 reversed the reduction of miR-146bi on anchorage-independent growth and monolayer growth, as compared with those observed in their scramble nonsense transfectants (**Figures 2C–E**).

Furthermore, the ectopic expression of HA-Cyclin D1 in UMUC3 (miR-146bi) cells also inhibited G0/G1 cell cycle arrest, and the results of flow cytometry also indicated that UMUC3 (miR-146bi/HA-Cyclin D1) exhibited a reverse effect of miR-146bi on inducing G0/G1 cell cycle arrest (**Figure 2F**). In conclusion, the results indicated that Cyclin D1 is a critical downstream effector for miR-146b mediating bladder cancer cell growth.

### 3.3 MiR-146b increased Cyclin D1 transcription by promoting C-MYC protein expression

**Figure 3:**
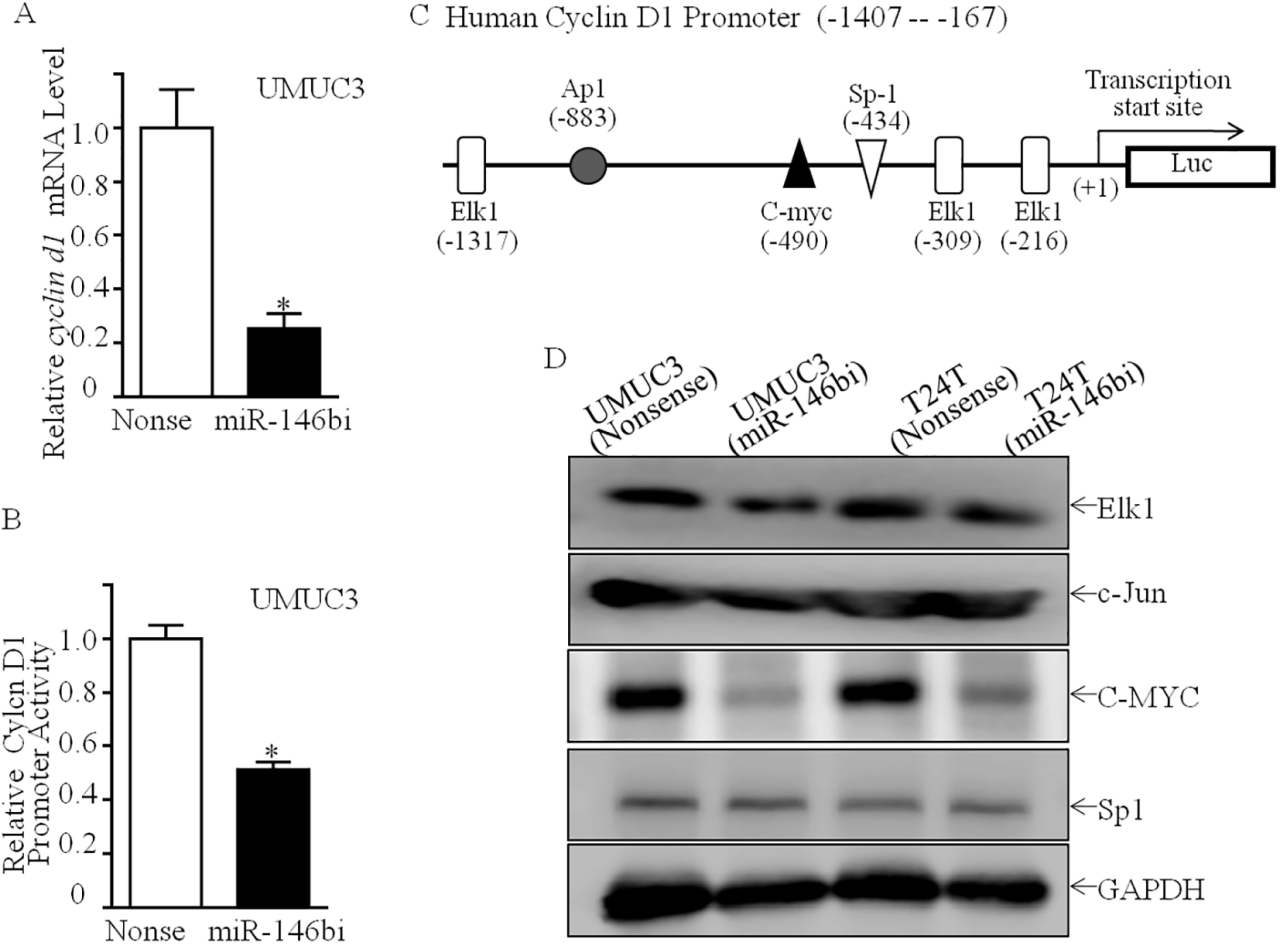
Inhibition of miR-146b attenuated Cyclin D1 transcription with downregulation of C-MYC protein. **(A)** Relative *cyclin d1* mRNA expression detected in UMUC3 (miR-146bi) and UMUC3 (nonsense) cells. **(B)** UMUC3 (nonsense) versus UMUC3 (miR-146bi) cells were transiently transfected with a *cyclin d1* promoter-driven luciferase reporter together with pRL-TK. Transfectants were seeded into 96-well plates to determine *cyclin d1* promoter transcriptional activity. pRL-TK was used as the internal control to normalize transfection efficiency. Bars indicate means ± SD from three replicate assays. **(C)** Potential transcriptional factor binding sites in human cyclin d1 promoter region were analyzed via the TRANSFAC 8.3 engine online. **(D)** Cell lysates from indicated cells were evaluated for Elk1, c-Jun, C-MYC, and Sp1 protein expression. GAPDH served as the loading control. The asterisk (*) indicates a statistically significant difference compared to the control (*p < 0.05).

To ascertain the mechanism of miR-146b upregulating Cyclin D1 expression, the mRNA expression of *cyclin* D1 was first assessed. As shown in **Figure 3A**, *cyclin d1* mRNA expression was profoundly downregulated in UMUC3 (miR-146bi) transfectant cells in comparison to UMUC3 (nonsense) cells. Subsequently, it was found that the Cyclin D1 promoter-driven luciferase reporter transcription activity in UMUC3 (miR-146bi) cells was much lower than in UMUC3 (nonsense) cells (**Figure 3B**), indicating the possibility of miR-146b increasing the transcription of *cyclin d1* mRNA. To verify this, we tried to conduct a bioinformatics analysis of the −1,407 to −167 region of the Cyclin D1 promoter sequence using the TFANSFAC Transcription Factor Binding Sites Software (Biological Database, Wolfenbüttel, Germany). The results showed that putative DNA-binding sites of Elk1, AP1, C-MYC, and Sp1 were involved in the Cyclin D1 promoter region (**Figure 3C**). Consequently, we tried to determine the effect of miR-146b inhibitor on the protein expression of the indicated transcription factors.

**Figure 4:**
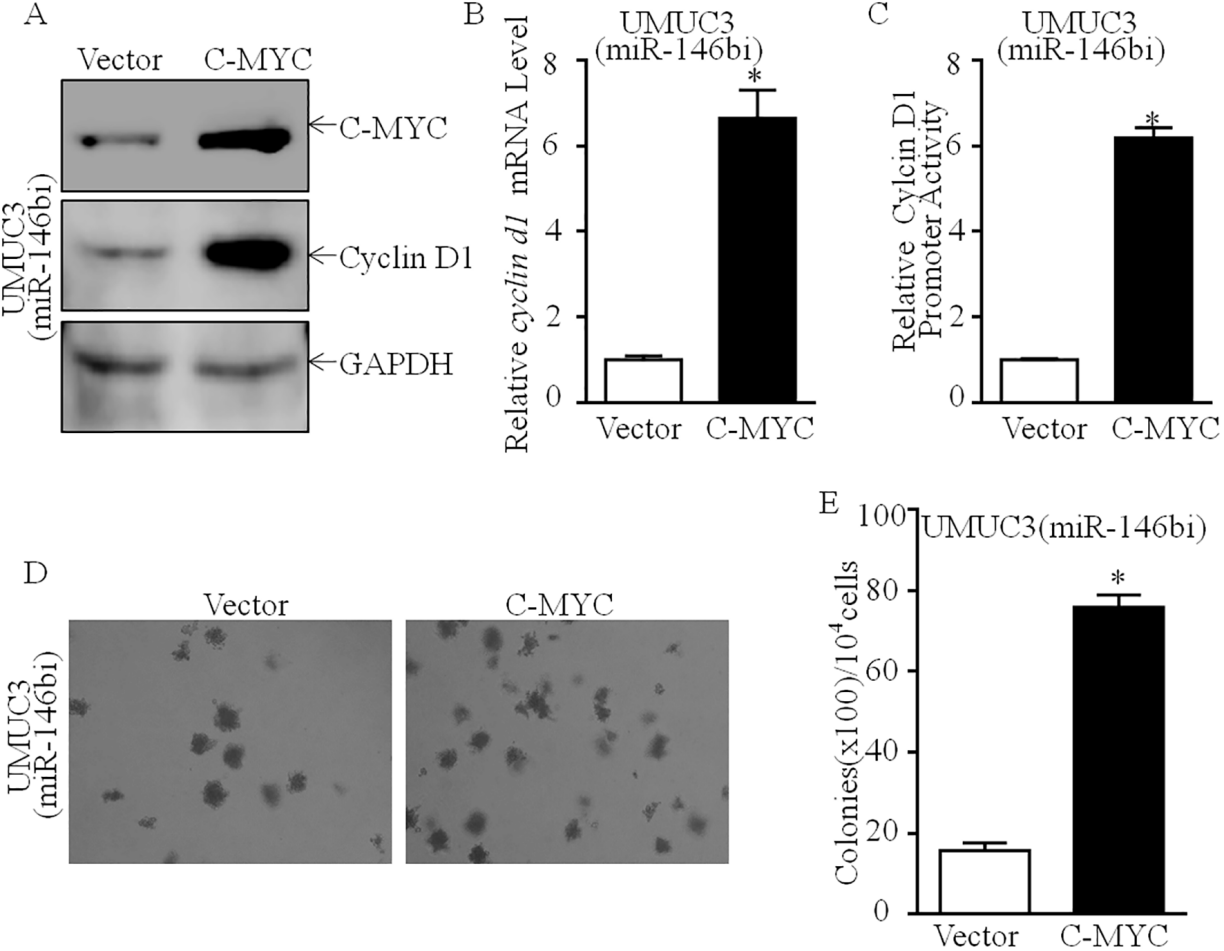
Effects of C-MYC on promotion of cyclin d1 transcription and human BCa anchorage-independent growth. **(A)** C-MYC overexpressed constructs were stably transfected into UMUC3 (miR-146bi) cells. The C-MYC overexpressed efficiency and its downstream Cyclin D1 expression were assessed by Western blotting. GAPDH was used as a protein loading control. **(B)** Relative *cyclin d1* mRNA expression detected in UMUC3 (miR-146bi/Vector) and UMUC3 (miR-146bi/C-MYC) cells. **(C)** UMUC3 (miR-146bi/Vector) and UMUC3 (miR-146bi/C-MYC) cells were transiently transfected with a *cyclin d1* promoter-driven luciferase reporter together with pRL-TK. Transfectants were seeded into 96-well plates to determine *cyclin d1* promoter transcriptional activity. pRL-TK was used as the internal control to normalize transfection efficiency. Bars indicate means ± SD from three replicate assays. **(D)** The stable transfectants as indicated were subjected to anchorage-independent soft agar growth assay. Representative images of colonies were photographed under an Olympus DP71. **(E)** The number of colonies was counted with more than 32 cells of each colony, and the results were presented as colonies per 10,000 cells. The bars show mean ± SD from three independent experiments, and the asterisk (*) indicates a significant increase in comparison to vector transfectants as indicated (*p < 0.05). BCa, bladder cancer.

As shown in **Figure 3D**, downregulation of C-MYC protein expression was discovered in UMUC3 (miR-146bi) cells, as compared to UMUC3 (nonsense) cells with no marked influence on Elk1, c-Jun, or Sp1 protein expression. Our previous study has shown that C-MYC is a promotive transcription factor of Cyclin D1 by directly binding to its promoter region, which plays a critical role in human bladder cancer cell tumorigenicity (19). Thus, we suggested that C-MYC may play a role in miR-146bi inhibition of Cyclin D1 transcription. To verify that C-MYC was critical for miR-146b promotion of human BCa cell proliferation, we transfected UMUC3 (miR-146bi) cells with the C-MYC-P3EF1a plasmid (**Figure 4A**). Consistently, the ectopic expression of C-MYC increased the mRNA expression and promoter activity of Cyclin D1, which remarkably increased the anchorage-independent growth ability of UMUC3 (miR-146bi/C-MYC) transfectants (**Figures 4B–E**). In conclusion, the above results strongly indicated that C-MYC is the key factor mediating miR-146b, increasing BCa growth by promoting Cyclin D1 transcription.

### 3.4 SMAD4 was required for miR-146b promotion of C-MYC at the transcriptional level

**Figure 5:**
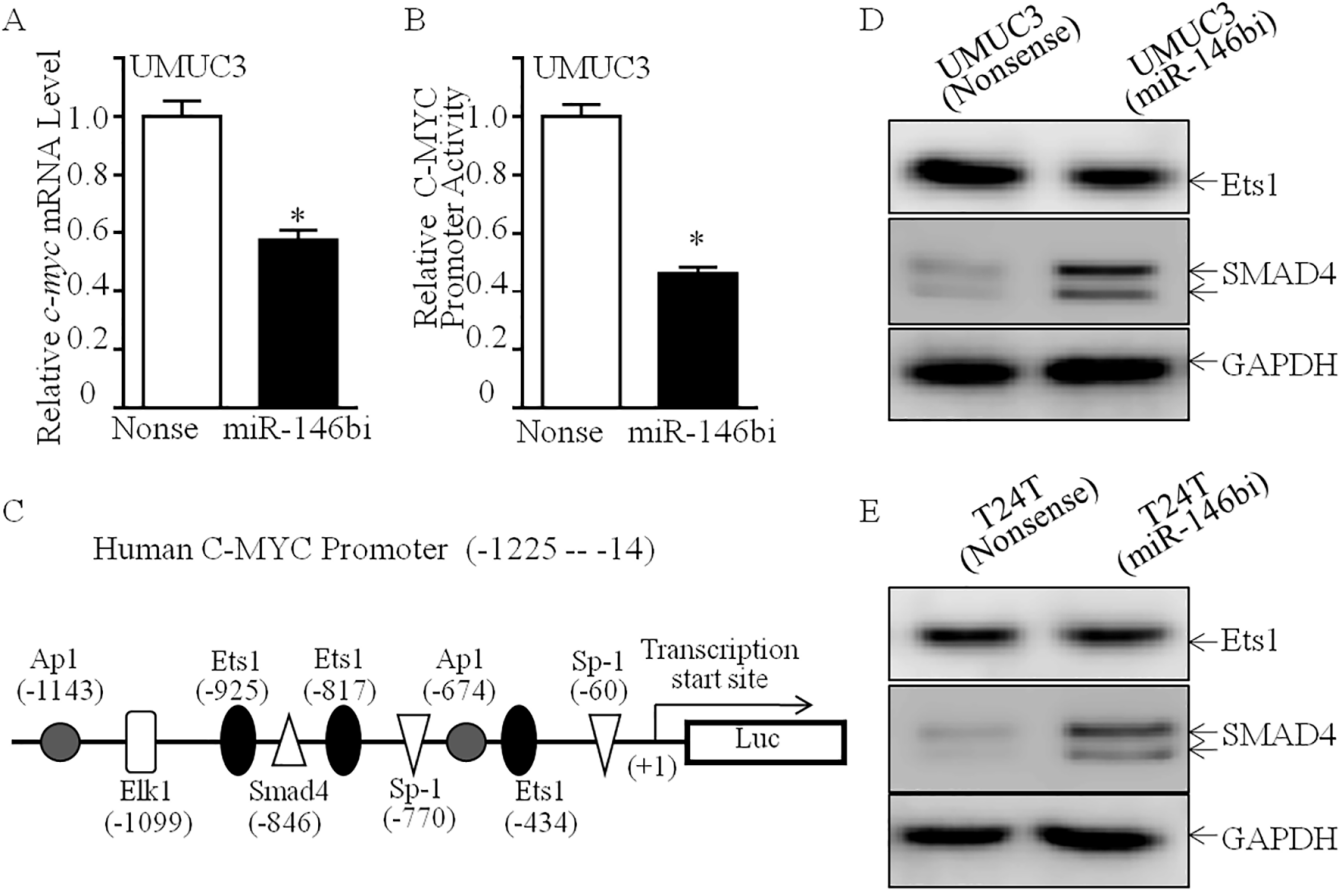
MiR-146b overexpression inhibited *c-myc* mRNA transcription in human BCa cells. **(A)** Relative *c-myc* mRNA expression detected in UMUC3 (miR-146bi) and UMUC3 (nonsense) cells. **(B)** UMUC3 (nonsense) versus UMUC3 (miR-146bi) cells were transiently transfected with a *c-myc* promoter-driven luciferase reporter together with pRL-TK. Transfectants were seeded into 96-well plates to determine *c-myc* promoter transcriptional activity. pRL-TK was used as the internal control to normalize transfection efficiency. Bars indicate means ± SD from three replicate assays. **(C)** Potential transcriptional factor binding sites in human *c-myc* promoter region were analyzed via the TRANSFAC 8.3 engine online. **(D, E)** Cell lysates from indicated cells were evaluated for Ets1 and SMAD4 protein expression. GAPDH served as the loading control. BCa, bladder cancer. The asterisk (*) indicates a statistically significant difference compared to the control (*p < 0.05).

**Figure 6:**
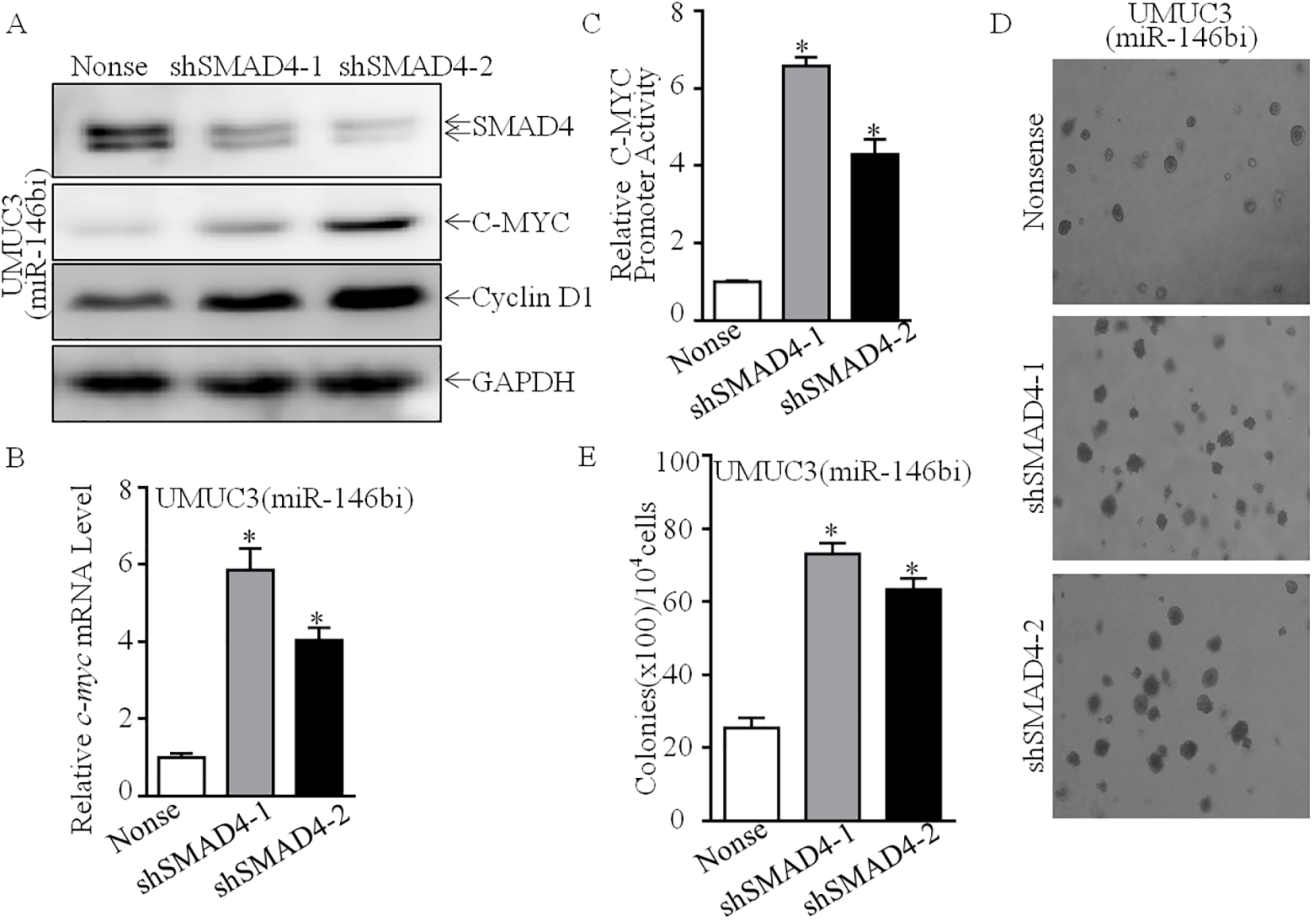
SMAD4 was a miR-146b-regulated transcription factor mediating Cyclin D1 upregulation in human BCa cells. **(A)** SMAD4 knockdown constructs were stably transfected into UMUC3 (miR-146bi) cells. The SMAD4 knockdown efficiency and its downstream C-MYC, Cyclin D1 expression were assessed by Western blotting. GAPDH was used as a protein loading control. **(B)** Relative *c-myc* mRNA expression detected in UMUC3 (miR-146bi/nonsense), UMUC3 (miR-146bi/shSMAD4-1) and UMUC3 (miR-146bi/shSMAD4-2) cells. **(C)** UMUC3 (miR-146bi/nonsense), UMUC3 (miR-146bi/shSMAD4-1), and UMUC3 (miR-146bi/shSMAD4-2) cells were transiently transfected with a *c-myc* promoter-driven luciferase reporter together with pRL-TK. Transfectants were seeded into 96-well plates to determine *c-myc* promoter transcriptional activity. pRL-TK was used as the internal control to normalize transfection efficiency. Bars indicate means ± SD from three replicate assays. **(D)** The stable transfectants as indicated were subjected to anchorage-independent soft agar growth assay. Representative images of colonies were photographed under an Olympus DP71. **(E)** The number of colonies was counted with more than 32 cells of each colony, and the results were presented as colonies per 10,000 cells. The bars show mean ± SD from three independent experiments, and the asterisk (*) indicates a significant increase in comparison to nonsense transfectants as indicated (*p < 0.05). BCa, bladder cancer.

In order to study the molecular mechanism of miR-146b upregulating C-MYC protein expression, we first tested the effect of miR-146b on *c-myc* mRNA level. We found that *c-myc* mRNA was significantly decreased in UMUC3 (miR-146bi) cells (**Figure 5A**). Furthermore, *c-myc* promoter-driven luciferase reporter activity was also inhibited in UMUC3 (miR-146bi) cells as compared with its scramble nonsense transfectants (**Figure 5B**), suggesting that the indicated transcription factor(s) involved in the −1,225 to −14 region of the promoter participated in miR-146b promoting *c-myc* promoter transcription. Therefore, we performed bioinformatics analysis to figure out the potential transcriptional factors (**Figure 5C**) and then detected their protein expression (Ap1, Elk1, Ets1, Smad4, and Sp1). The results indicated that only SMAD4 showed high expression, while others exhibited no remarkable differences (**Figures 3D**, **5D, E**), revealing that SMAD4 may be critical for regulating C-MYC expression. Thus, we knocked down SMAD4 in UMUC3 (miR-146bi) cells using shRNAs specifically targeting human SMAD4, and UMUC3 (miR-146bi/shSMAD4#1), UMUC3 (miR-146bi/shSMAD4#2), and their scramble transfectant UMUC3 (miR-146bi/nonsense) were established (**Figure 6A**). As expected, the knockdown of SMAD4 increased the protein expression of C-MYC and Cyclin D1 (**Figure 6A**), *c-myc* mRNA expression (**Figure 6B**), and *c-myc* promoter activity (**Figure 6C**), as well as BCa cell growth (**Figures 6D, E**), demonstrating that SMAD4 was indeed an intermediate regulator linking miR-146b to C-MYC and responsible for BCa tumorigenic growth. In general, miR-146 inhibits SMAD4 expression, consequently increasing *c-myc* transcription, further promoting *cyclin d1* mRNA transcription and protein expression, and finally elevating BCa cell anchorage-independent growth.

### 3.5 MiR-146b decreased *smad4* mRNA stability by directly targeting its 3′-UTR

**Figure 7:**
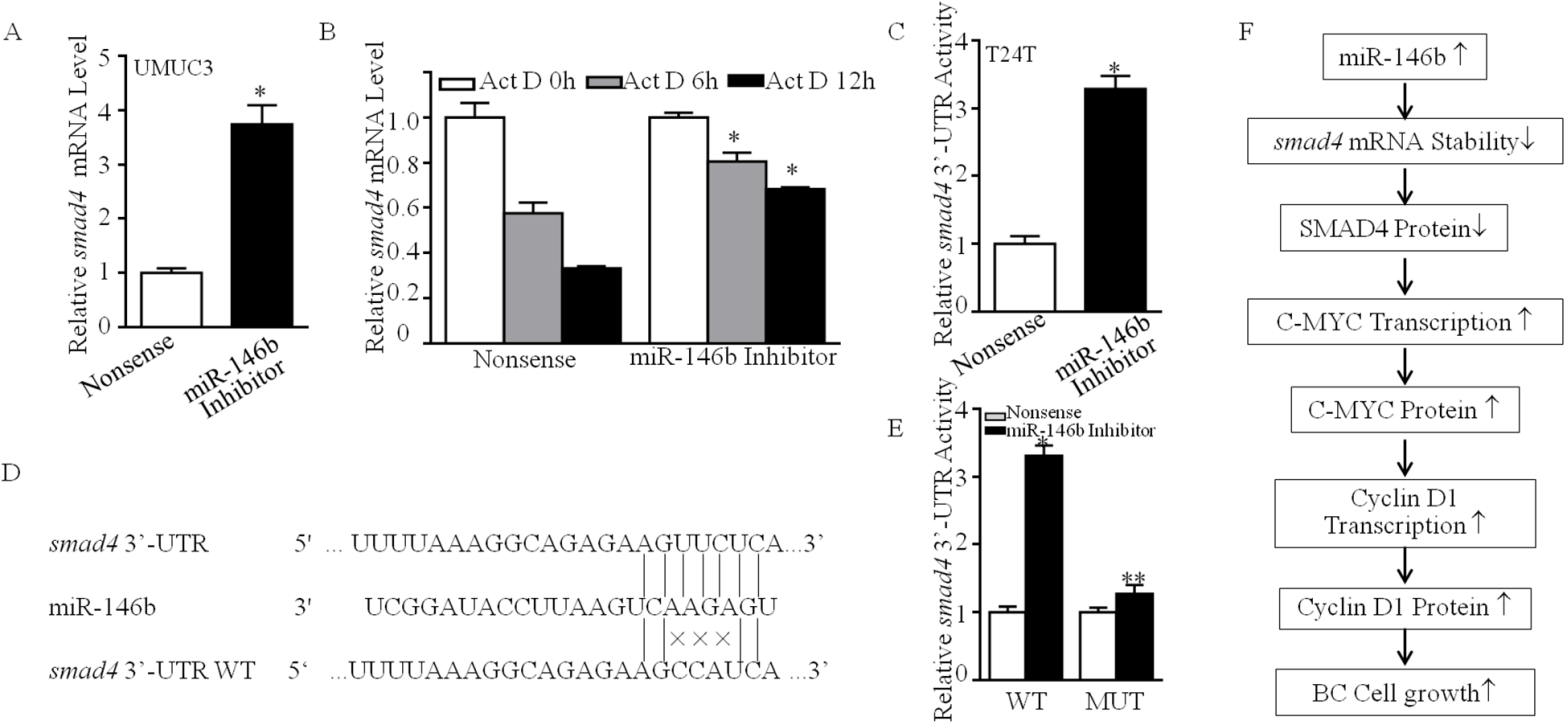
MiR-146b was responsible for destabilization of *smad4* mRNA by direct binding to its 3′-UTR. **(A)** UMUC3 (miR-146bi) and UMUC3 (nonsense) cells were cultured in 6-well plates till cell density reached 70%–80%. Following synchronization for 12 h, the medium was then replaced with 10% FBS DMEM for another 12 h The cells were extracted for total RNA with TRIzol reagent. Real-time PCR was used to determine *smad4* mRNA expression, and gapdh was used as an internal control. Data represent mean ± SD (*p < 0.05). **(B)** UMUC3 (miR-146bi) and UMUC3 (nonsense) cells were seeded into 6-well plates. After synchronization, the indicated cells were treated with Actinomycin D (Act D) for the indicated time points. Total RNA was then isolated and subjected to real-time PCR analysis for mRNA levels of *smad4*, and *gapdh* was used as an internal control. Bars represent mean ± SD from three independent experiments. Student’s *t*-test was utilized to determine the p-value. The asterisk (*) indicates a significant increase in comparison to UMUC3 (nonsense) cells (*p < 0.05). **(C)** The pMIR-*SMAD4 3′-UTR* reporter was transiently transfected into the indicated cells, and luciferase activity of each transfectant was evaluated. The luciferase activity was presented as a relative to nonsense transfectant normalized using pRL-TK as an internal control. The bars show mean ± SD from three independent experiments. The symbol (*) indicates a significant increase in UMUC3 (miR-146bi) in comparison to nonsense transfectant (p < 0.05). **(D)** The predicted miR-146b binding site existed in the 3′-UTR of *smad4* mRNA, and its mutants (MUTs) were generated in the binding site. **(E)** The pMIR-*smad4* 3′-UTR reporters (WT and MUT) were co-transfected with pRL-TK into the indicated cells. Twenty-four hours post-transfection, the transfectants were extracted for determination of the luciferase activity, and TK was used as the internal control. Luciferase activity of each transfectant was evaluated, and the results were presented as relative *SMAD4 3′-UTR* activity. The bars show mean ± SD from three independent experiments. The double asterisk (**) indicates a significant inhibition of *3′-UTR* activity in mutant transfectant in comparison to mutant of WT *smad4 3′-UTR* luciferase reporter transfectant (p < 0.05). **(F)** The proposed mechanisms underlying miR-146b overexpression in the promotion of human BCa cells growth: miR-146b overexpression destabilizes *smad4* mRNA, which further elevates *c-myc* mRNA transcription, in turn promoting the transcription of *cyclin d1* and protein expression, and finally increases the transition of cell cycle G0/G1 phase and the anchorage-independent growth of human BCa cells. FBS, fetal bovine serum; DMEM, Dulbecco’s modified Eagle’s medium; BCa, bladder cancer.

To clarify the molecular mechanism of miR-146b inhibiting SMAD4 expression, we first detected *smad4* mRNA abundance in UMUC3 cells. As shown in **Figure 7A**, miR-146b inhibition elevated *smad4* mRNA expression. Therefore, we supposed that miR-146b destabilized *smad4* mRNA. The result exhibited that miR-146b inhibition delayed the *smad4* mRNA degradation rates (**Figure 7B**). MiRNAs function post-transcriptionally usually by base-pairing to target mRNA 3′-UTR to inhibit protein synthesis (20). Thus, we next detected the 3′-UTR activity of s*mad4* mRNA between UMUC3 (miR-146bi) and UMUC3 (nonsense) cells. As shown in **Figure 7C**, miR-146b inhibition improved the *smad4* mRNA 3′-UTR activity as compared to the control cells, suggesting that miR-146b may be involved in this regulation. Thereby, we performed bioinformatics research on the potential targeted miRNA of smad4 mRNA 3′-UTR using TargetScan (v7.0; targetscan.org) (21). The results exhibited that there were multiple putative miRNA binding sites in *smad4* mRNA 3′-UTR, surprisingly including the binding sites for miR-146b (**Figure 7D**).

To ascertain whether miR-146b could directly bind to *smad4* mRNA 3′-UTR to stabilize its mRNA, as shown in **Figure 7D**, we constructed the plasmid that miR-146b binding site in *smad4* mRNA 3′-UTR-luciferase reporter was mutated. Then, we co-transfected the wild-type or the mutation plasmids with pRL-TK into either UMUC3 (nonsense) or UMUC3(miR-146bi) cells transiently. We found that the luciferase activity of wild-type *smad4* mRNA 3′-UTR in UMUC3 (miR-146bi) cells was much higher than in UMUC3 (nonsense), whereas the mutation decreased the effect of miR-146b inhibition (**Figure 7E**), revealing that the direct binding of miR-146b to *smad4* mRNA 3′-UTR is critical for miR-146b stabilization of *smad4* mRNA. In summary, our results revealed that overexpressed miR-146b attenuated *smad4* mRNA expression by directly binding to its 3′-UTR, therefore promoting *c-myc* mRNA transcription, in turn increasing *cyclin D1* mRNA transcription and protein expression and finally promoting BCa cell anchorage-independent growth as shown in **Figure 7F**.

## 4 Discussion

In recent years, accumulating evidence demonstrated microRNAs as negative regulators of gene expression by directly binding with mRNA sequences to repress transcript destabilization or translation (22, 23). A growing number of studies have shown that the dysregulation of miRNAs leads to the progression of multiple cancers, including bladder cancer (24). Nevertheless, the aberrant expression of specific miRNAs exerting the actual functions on proliferation, metastasis, and progression of BCa is still unclear. MiR-146b is a member of the miR-146 family, which is encoded by independent genes located on human chromosomes 10 (25). Several studies have reported that miR-146b is widely upregulated and plays an important oncogenic role in multiple cancers, such as cervical cancer (7), colon cancer (8), and thyroid cancer (26). Our previous data illustrate that the drastic upregulation of miR-146b in BCa tissues and cell lines was discovered as expected, as compared to its adjacent normal urothelial tissues and the normal urothelial cell line, respectively. Moreover, miR-146b overexpression promotes the invasion ability of human bladder cancer by increasing MMP2 protein expression (10). However, its role in the malignant growth and proliferation of BCa has not been fully studied. In this research, the loss-of-function study demonstrated that the inhibition of miR-146b could repress the proliferation and anchorage-independent growth of BCa cells, which suggested miR-146b as an oncogene in BCa. While our data suggest that miR-146b directly regulates the SMAD4/C-MYC/Cyclin D1 axis, it is important to note that miR-146b may not be the only regulator of this pathway. Other miRNAs and transcription factors could also influence the expression of SMAD4, C-MYC, and Cyclin D1, further modulating bladder cancer cell growth and proliferation. For instance, miRNAs such as the miR-17-92 cluster and miR-26a have been implicated in regulating components of the SMAD4/C-MYC/Cyclin D1 axis in other cancer types. Future studies focusing on the combined effects of miR-146b and other regulatory elements may offer a more comprehensive understanding of the intricate regulatory networks governing bladder cancer progression.

Our further study indicated that the G0/G1 phase arrest could be responsible for miR-146b inhibition inducing cell proliferation impairment. Cyclin D1, a critical regulator, controls the cell cycle progression from the G0–G1 phase to the S phase by forming a complex with CDK4/6 (27, 28). Deregulation of Cyclin D1 expression and/or activity is common in human cancers including bladder cancer (29), colon cancer (30), and breast cancer (31). Cyclin D1 has been reported to be responsible for miR-576-3p overexpression inhibiting human bladder cancer proliferation by directly binding with its 3′-UTR (32). Isorhapontigenin exerts cancer-inhibitory effects on the growth of patient-derived glioblastoma spheres by inhibiting Cyclin D1 expression by regulating the miR-145/SOX2 axis (33). Long non-coding RNA (lncRNA) DILA1 could interact with the Thr286 of Cyclin D1 for repressing its phosphorylation and subsequent degradation, thereby promoting the Cyclin D1 protein expression and finally contributing to tamoxifen resistance in breast cancer (34). In the current study, miR-146b inhibition decreased Cyclin D1 protein expression. Ectopically expressed HA-Cyclin D1 in UMUC3 (miR-146bi) cells expectably reversed the reduction of miR-146bi on monolayer and anchorage-independent growth, indicating that Cyclin D1 is responsible for miR-146bi-mediated G0/G1 cell cycle arrest and the inhibition of anchorage-independent growth in BCa cells.

Oncoprotein C-MYC is critical for the formation and progression of various cancers by triggering the transcription of downstream oncogene or tumor suppressor genes (35). C-MYC could collaborate with TGF-α, epidermal growth factor receptor, Ras, PI3K/Akt, or NF-κB to the regulation of Cyclin D1 (36, 37). Coordination of C-MYC with Cyclin D1 not only accelerates tumor formation but also may drive tumor progression to a more aggressive phenotype (38). Our previous study has shown that *c-myc* mRNA impairment is crucial for p63α inhibition of Cyclin D1 gene transcription and bladder cancer cell tumorigenicity (19). In the present study, we explored the mechanism underlying miR-146b promoting Cyclin D1 promoter activity and focused on the contribution of C-MYC on Cyclin D1 promoter activity. We found that miR-146b inhibition could reduce Cyclin D1 mRNA expression through the inhibition of its promoter activity, which is mediated by decreasing C-MYC protein expression. Moreover, the ectopic expression of C-MYC reversed not only the inhibition effect on Cyclin D1 expression but also the anchorage-independent growth by knockdown miR-146b in BCa cells. Taken together, our study provides evidence that targeting C-MYC and Cyclin D1 may be a good strategy for bladder cancer prevention.

Heterozygous or homozygous deletion of SMAD4 was first discovered in pancreatic ductal adenocarcinoma (39) and later detected in various types of cancers, such as colorectal cancer (40), cholangiocarcinoma (41), gastric cancer (42), and prostate cancer (43), although with lower frequencies to some extent. In bladder cancer, SMAD4 overexpression decreased bladder cancer cell proliferation, migration, and invasion abilities (44). Herein, the knockdown of miR-146b stabilized *smad4* mRNA and promoted its protein expression by directly binding with its 3′-UTR. Knockdown of SMAD4 in UMUC3 (miR-146bi) cells reversed the inhibition of anchorage-independent growth of BCa, indicating the consistent suggestion that SMAD4 may be a tumor suppressor for BCa tumorigenic growth. As the core mediator of the canonical TGF-β signaling pathway, SMAD4 plays a pivotal role in the switch of TGF-β function on tumorigenesis (45). The canonical TGF-β/SMAD4 signaling pathway plays a tumor suppressive role at early stages, mainly by inducing G0/G1 cell cycle arrest and apoptosis (46). Moreover, SMAD4 mediates the inhibitory effect of TGF-β on C-MYC expression by forming a complex with SMAD3, E2F4/5, and p107 at the TGF-β inhibitory element (TIE) element on the *c-myc* promoter (47). Consistently, in this study, we found that the knockdown of SMAD4 in UMUC3 (miR-146bi) cells promoted C-MYC transcription and protein expression. The results of the ChIP assay showed that SMAD4 could directly bind with the *c-myc* promoter.

Our findings provide novel insights into the role of miR-146b in regulating the SMAD4/C-MYC/Cyclin D1 axis in bladder cancer, aligning with previous studies demonstrating miR-146b’s oncogenic potential in various malignancies. In colorectal cancer, miR-146b has been shown to regulate cancer cell proliferation and invasion, similarly enhancing the transcription of Cyclin D1 and other cell cycle regulators through its modulation of key signaling pathway state cancer; miR-146b also promotes cell growth by targeting PTEN and activating the PI3K/AKT pathway, which can intersect with the mechanisms we can observe in bladder cancer. In cervical cancer, miR-146b enhances proliferation and metastasis by regulating inflammatory pathways, a mechanism that could potentially overlap with its effects on the TGF-β/SMAD signaling axis in bladder cancer. These studies and our findings underscore the central role of miR-146b in promoting cancer progression through the regulation of cell cycle-related proteins such as Cyclin D1, C-MYC, and other pivotal regulators. However, it is important to recognize that miR-146b may not be the only regulator of this axis. Other miRNAs, such as the miR-17-92 cluster, have been implicated in modulating the same pathway in different cancers. Future research should explore the cooperative regulation of this axis by multiple miRNAs and transcription factors, providing a more comprehensive understanding of the regulatory networks involved in bladder cancer progression. Although our study provides valuable insights into the role of miR-146b in bladder cancer, there are several limitations that should be acknowledged. First, our study mainly relied on the use of the UMUC3 and T24T cell lines and athymic nude mice, which may not fully recapitulate the complexity of human bladder cancer, especially with regard to tumor heterogeneity and the tumor microenvironment. Furthermore, while our findings suggest a significant role for miR-146b in regulating the SMAD4/C-MYC/Cyclin D1 axis, other potential pathways or factors may also contribute to the observed effects. Future research should explore additional bladder cancer models, including PDXs or GEMMs, to better reflect tumor biology and therapeutic responses. Additionally, the potential off-target effects of miR-146b inhibition were not fully addressed in this study, and future work should include controls to evaluate these effects more comprehensively. Lastly, while we focused on the miR-146b-induced regulation of Cyclin D1 and C-MYC, additional miRNAs or transcription factors may cooperate in modulating this axis, warranting further investigation into the broader regulatory network involved in bladder cancer progression.

While our study highlights miR-146b as a promising therapeutic target in bladder cancer, it is important to consider the challenges associated with miRNA-based therapies. MiRNA therapies face several obstacles, including the efficient delivery of miRNA mimics or inhibitors to target tissues, off-target effects, and potential immune responses. Additionally, the systemic delivery of miRNAs is often hindered by their instability in the bloodstream and rapid degradation by nucleases. Recent advancements in nanotechnology, such as liposomes and nanoparticles, hold promise for improving the targeted delivery and stability of miRNA-based therapies. Furthermore, the specificity of miRNA inhibition or overexpression can be fine-tuned by employing modified RNA molecules with reduced off-target effects. Therefore, while miR-146b-based therapies show potential, future research should focus on optimizing delivery methods, minimizing off-target effects, and assessing the safety and efficacy of miRNA-based approaches in preclinical and clinical trials.

In conclusion, miR-146b overexpression promotes proliferation and anchorage-independent growth of human BCa cells by enhancing SMAD4/C-MYC/Cyclin D1 pathway activation. MiR-146b overexpression destabilizes *smad4* mRNA by directly binding to its 3′-UTR, further elevating *c-myc* mRNA transcription, followed by promoting *cyclin D1* mRNA transcription and protein expression, and finally promoting the transition of cell cycle G0/G1 phase and the anchorage-independent growth of human BCa cells. The observed regulation of the SMAD4/C-MYC/Cyclin D1 axis by miR-146b underscores its potential as a key modulator in bladder cancer progression. However, it is essential to recognize that this pathway may be influenced by other factors beyond miR-146b. Other miRNAs, such as the miR-17-92 cluster, have been shown to target SMAD4 and C-MYC in different cancers, suggesting that multiple miRNAs may cooperatively regulate the same pathway. Additionally, transcription factors and signaling molecules such as TGF-β and SMAD2/3 may also play roles in modulating this axis. Further investigation into these alternative regulatory mechanisms will help provide a more complete picture of the molecular drivers of bladder cancer and may reveal additional therapeutic targets. Our new findings together with previous data of miR-146b promoting bladder cancer invasion suggest that the miR-146b could serve as a promising prognostic and therapeutic target for bladder cancer management.

## 5 Conclusions

In conclusion, our study demonstrates that miR-146b plays a crucial role in promoting the proliferation and tumorigenic growth of BCa cells through the modulation of the SMAD4/C-MYC/Cyclin D1 axis. Specifically, miR-146b overexpression leads to the destabilization of SMAD4 mRNA by directly binding to its 3’-UTR, thereby decreasing SMAD4 expression. This reduction in SMAD4 facilitates the transcription of C-MYC, which, in turn, upregulates Cyclin D1 expression, driving the G0/G1 cell cycle transition and enhancing BCa cell proliferation. Furthermore, our findings highlight that miR-146b not only is involved in the regulation of key cell cycle regulators but also plays a significant role in promoting anchorage-independent growth, a critical hallmark of tumorigenic potential. The therapeutic implications of these findings are noteworthy, as targeting miR-146b could provide a novel strategy for controlling BCa cell growth by disrupting this oncogenic pathway.

This study presents a new mechanistic insight into the role of miR-146b in bladder cancer and its potential as a therapeutic target. Given that miR-146b has already been implicated in various cancer types, its involvement in BCa suggests that it may serve as both a biomarker for prognosis and a target for therapeutic intervention. Future studies should focus on validating these findings in clinical settings and exploring potential miR-146b-based therapies for BCa treatment.

## Data Availability

The original contributions presented in the study are included in the article/supplementary material. Further inquiries can be directed to the corresponding authors.
